# Quantification of cooling effects on basic tissue measurements and exposed cross-sectional brain area of cadaver heads from market pigs

**DOI:** 10.1093/tas/txab001

**Published:** 2021-01-11

**Authors:** Karly N Anderson, Sarah E Albers, Kaysie J Allen, Katherine D Bishop, Brian J Greco, Christina M Huber, Ashlynn A Kirk, Hannah Olsen, Kurt D Vogel

**Affiliations:** 1 Department of Veterinary Population Medicine, College of Veterinary Medicine, University of Minnesota, St. Paul, MN, USA; 2 Department of Animal and Food Science, University of Wisconsin–River Falls, River Falls, WI, USA; 3 Department of Animal and Dairy Sciences, University of Wisconsin–Madison, Madison, WI, USA; 4 Center for Animal Welfare, Department of Animal Science, University of California–Davis, Davis, CA, USA

**Keywords:** captive bolt, euthanasia, slaughter, stunning, swine

## Abstract

The objective of this project was to determine the impact of cooling on the soft tissue thickness, cranial thickness, and cross-sectional brain area of cadaver heads from market pigs. Documenting the effect of cooling on tissue dimensions of swine heads is valuable and important for future investigations of physical stunning and euthanasia methods that use cadaver heads. Scalded and dehaired cadaver heads with intact jowls were sourced from market pigs stunned with CO_2_ gas. After transport to the data collection location, a penetrating captive bolt (PCB) shot (Jarvis Model PAS—Type P 0.25R Caliber Captive Bolt Pistol with Medium Rod Assembly and Blue Powder Cartridges) was applied in the frontal position. Following PCB application, each head (*n* = 36) underwent an UNCHILLED treatment followed by CHILLED treatment. The UNCHILLED treatment involved images collected immediately after splitting each head along the bolt path, and the CHILLED treatment involved images of the same heads after storage in a walk-in cooler for 24 h at 2 to 4°C. All measurements for each treatment were collected from images of the heads on the plane of the bolt path immediately prior to and immediately after the refrigeration treatment. Measurements were performed by two observers. Across all measurements, mean interobserver coefficient of variation was 11.3 ± 0.6%. The soft tissue caudal to the bolt path was different (*P* = 0.0120) between treatments (CHILLED: 6.4 ± 0.2 mm; UNCHILLED: 7.2 ± 0.2 mm). The soft tissue thickness rostral to the bolt path was different (*P* = 0.0378) between treatments (CHILLED: 5.5 ± 0.2 mm; UNCHILLED: 6.1 ± 0.2 mm). Cranial thickness caudal to the bolt path was not different (*P* = 0.8659; CHILLED: 18.1 ± 0.6 mm; UNCHILLED: 18.3 ± 0.6 mm), nor was there a significant difference (*P* = 0.2593) in cranial thickness rostral to the bolt path between treatments (CHILLED: 16.2 ± 0.6 mm; UNCHILLED: 15.2 ± 0.6 mm). Cross-sectional brain area did not differ (*P* = 0.0737; CHILLED: 3633.4 ± 44.1 mm; UNCHILLED: 3519.9 ± 44.1 mm). A correction factor of 1.12 was determined from this study for cases where estimation of UNCHILLED soft tissue thickness from CHILLED soft tissue thickness is necessary.

## INTRODUCTION

Penetrating captive bolt (PCB) is a method of euthanasia and stunning approved for a variety of livestock species by the American Veterinary Medical Association (AVMA 2016, 2020), the National Pork Board (NPB) and American Association of Swine Veterinarians (AASV) for swine (NPB, 2016), and the American Association of Bovine Practitioners (AABP, 2019) for cattle. There has been growing interest in assessing the implications of bolt length used with cattle (Kline et al., 2019; Wagner et al., 2019), physical landmarks for PCB placement for cattle (Gilliam et al., 2012, 2016, 2018), and alternative PCB placement in swine (Anderson et al., 2019). The brain is a semifluid structure that makes the assessment of brain damage attributed to the PCB itself difficult due to tissue distortion during the head splitting process (Kline et al., 2019). At the time of our study, there did not appear to be any published data to quantify the impact of cooling on the tissue parameters of cadaver heads of any species following the application of a captive bolt. Understanding and accounting for potential changes in tissue dimensions associated with cooling are important for future investigations related to the captive bolt stunning and euthanasia of livestock species, as chilling allows for a greater degree of accuracy when assessing brain damage because the bolt path remains clearly visible in cooled brain tissue.

The objective of this study was to quantify the impact of cooling on the soft tissue thickness, cranial thickness, total tissue thickness, and cross-sectional brain area of cadaver heads from market pigs following the application of a PCB shot to the common frontal location. Our hypothesis was that UNCHILLED cadaver heads would display soft tissue thickness, cranial thickness, total tissue thickness, and cross-sectional brain area that was not different than CHILLED cadaver heads.

## MATERIALS AND METHODS

### Animal Use Protocol

It was not necessary to submit an animal use protocol to the University of Wisconsin—River Falls Institutional Animal Care and Use Committee (IACUC) because live animals were not directly manipulated in this study. The pigs from which the heads were obtained were slaughtered at a commercial slaughter establishment under inspection by the United States Department of Agriculture Food Safety and Inspection Service (USDA FSIS) in accordance with the regulations in 9 CFR 313. The heads used in this study were acquired as meat products that were suitable for human consumption. The exemption from IACUC approval followed the precedent established by Anderson et al. (2019).

### Description of Cadaver Heads

Thirty-six heads, scalded and dehaired with skin on and jowls intact, were obtained from pigs that were commercially slaughtered at a regional processing facility under federal inspection. The heads were inspected and passed for human consumption. The estimated body weight (**BW**) of the pigs was 136 kg, and they were approximately 6 mo of age. Exact BW and age of each pig was not available, so estimates of age and weight were based on the purchasing specifications of the establishment. Each head was removed from its respective carcass via knife incision between the atlas and axis by plant personnel. All heads were packaged in plastic bags and boxed prior to unrefrigerated transport (distance traveled: 180 km) to the University of Wisconsin—River Falls Meat Science Laboratory where the project commenced within approximately 6 h of head collection.

### Description of Captive Bolt Tool and Placement

The PCB device used in this study was a Jarvis Model PAS—Type P 0.25R Caliber Captive Bolt Pistol (order no. 4144035, Jarvis Corp., Middletown, CT) equipped with Medium Stunning Rod Nosepiece Assembly (order no. 3116604, Jarvis Corp.). Jarvis Blue Powder Cartridges—0.25R Caliber, 3GR (order no. 1176018, Jarvis Corp.) were utilized for all PCB applications in this study. All equipment and placement procedures were the same as used by Anderson et al. (2019). The PCB was placed in the frontal location as described by Woods et al. (2010) at 2.54 cm superior to a line drawn across the top of the eyes at the midline. To ensure consistent placement and entry angle of the PCB, the barrel of the captive bolt pistol was fitted with a custom-fabricated flange. The flange maintained a perpendicular relationship between the PCB and frontal plate of the cranium. To prevent the movement during PCB application, each head was placed on a solid surface and held firmly in place by the snout and the ears.

### Postapplication Head Processing

Following the application of the shot location treatment, each head was cut along the bolt path with a meat band saw. Following each cut, digital images were collected from both the left and right sides of each exposed intracranial surface. Thermal images (Model E8, FLIR Systems, Boston, MA) were also collected from both the left and right sides of each exposed intracranial surface for temperature assessment. All images were collected with the thermal camera and digital camera positioned 54.6 cm directly above and perpendicular to the exposed cut surface. Following image collection, the two halves of each head were reassembled and wrapped with polyvinylchloride film prior to chilling for 24 h at 2 to 4°C.

### Thermal Image Collection and Analysis

Temperature data were collected with a thermal imaging camera (FLIR E8, FLIR Systems). The accuracy of temperature emission readings for this model was ±2°C or ±2% of reading, for ambient temperature 10 to 35°C and object temperature above 0°C. The camera also utilized Multi-Spectral Dynamic Imaging to enhance the thermal image with a visible camera detail based on a simultaneously collected digital image. Thermal images were uploaded to a laptop computer and viewed on FLIR Tools software (FLIR Systems) to determine maximum intracranial temperature. All digital images were also uploaded to a laptop computer for tissue and area measurement collection.

### Tissue and Cranial Measurements

**Figure 1.**
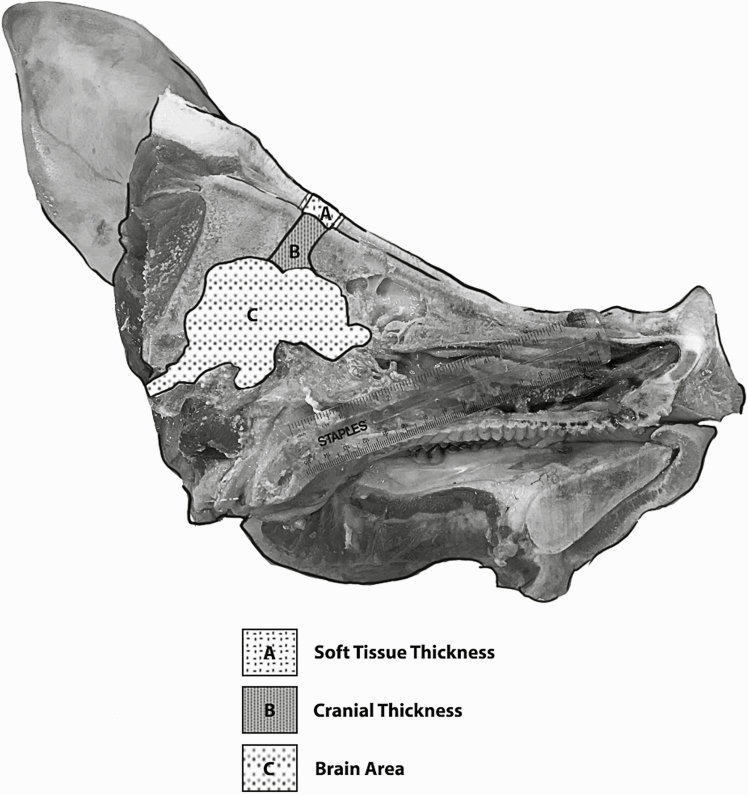
Soft tissue thickness—the tissue from the application site to the exterior surface of the cranium along the bolt path; cranial thickness—the thickness from the exterior surface of the cranium to the interior surface of the cranium along the bolt path; brain area—the cross-sectional surface area of the exposed brain area within the plane of the bolt travel. Soft tissue thickness and cranial thickness were measured at the rostral and caudal aspects of the bolt path on the right and left halves of each split head. Values were averaged prior to statistical analysis.

Measurements of soft tissue thickness (mm), cranial thickness (mm), and cross-sectional brain area (mm^2^; Figure 1) were determined from images collected at the time of head processing for the control heads and 24 h after head processing for chilled heads. All images included a 15.0-cm ruler that was used as a reference for an online irregular area calculator (SketchandCalc, iCalc, Inc., Palm Coast, FL). All measurements were performed by two trained observers. Across all measurements, the mean interobserver percent coefficient of variation was 11.3 ± 0.6%. Soft tissue thickness referred to the tissue from the application site to the exterior surface of the cranium. This measurement was reported caudal and rostral to the bolt path. Cranial thickness referred to the thickness from the exterior surface of the cranium to the interior surface of the cranium along the bolt path. This measurement was reported caudal and rostral to the bolt path. Total tissue thickness (mm), which referred to the total soft tissue and cranial thickness from the site of application to the interior surface of the cranium, was determined from the summation of the soft tissue and cranial thicknesses for each cadaver head. This measurement was reported caudal and rostral to the bolt path. Soft tissue thickness, cranial thickness, and total tissue thickness were determined by averaging the measurements from the right and left halves each head for the rostral aspect and the caudal aspect of the bolt path. Cross-sectional brain area (mm^2^) referred to the cross-sectional surface area of the exposed brain area within the plane of the bolt travel as described by Anderson et al. (2019). Measurements of cross-sectional brain area were calculated from both the right and left halves along the paths of bolt travel and averaged prior to statistical analysis. All measurements were repeated 24 h after each head was placed under refrigeration.

### Statistical Analyses

All continuous data for captive bolt application treatment (UNCHILLED, CHILLED) effects were analyzed using models constructed within the MIXED procedure of SAS (Statistical Analysis System Institute, Inc., Cary, NC) with Kenward–Roger denominator degrees of freedom designated within each model and mean separation performed with the Tukey’s test designation. For all analyses, the experimental unit was individual head. Significant differences in treatment effects were recognized at α ≤ 0.05.

## RESULTS AND DISCUSSION

Tissue measurements, cross-sectional brain areas, weights, and temperatures collected from this study can be observed in Table 1. Soft tissue thickness was significantly different (*P* < 0.05) between the UNCHILLED and CHILLED treatments. There were no significant differences (*P* > 0.05) in cranial thickness, total tissue thickness, and cross-sectional brain area between the UNCHILLED and CHILLED treatments. Head weight was not significantly different (*P* > 0.05) between the UNCHILLED and CHILLED treatments. Cranial temperature was significantly different (*P* < 0.05) between the UNCHILLED and CHILLED treatments.

**Table 1.** Effects of cooling on tissue parameters and cross-sectional brain area of cadaver heads from market weight hogs assigned to an UNCHILLED and CHILLED treatment and sectioned by band saw following the plane of bolt travel (*n* = 36)

	Refrigeration treatment^1^					
	UNCHILLED		CHILLED			
Dependent variable	SEM	*n*	SEM	*n*	Pooled SE	*P*-value
Soft tissue thickness caudal to bolt path, mm	7.2	36	6.4	36	0.2	0.0120
Soft tissue thickness rostral to bolt path, mm	6.1	36	5.5	36	0.2	0.0378
Cranial thickness caudal to bolt path, mm	18.3	35	18.1	36	0.6	0.8659
Cranial thickness rostral to bolt path, mm	15.2	36	16.2	36	0.6	0.2593
Total tissue thickness caudal to bolt path, mm	24.5	35	24.5	36	0.6	0.2577
Total tissue thickness rostral to bolt path, mm	21.3	36	21.7	36	0.7	0.6730
Cross-sectional brain area, mm^2^	3519.9	30	3633.4	31	44.1	0.0737
Head weight, kg	5.8	36	5.6	36	0.1	0.1824
Exposed head temperature, °C	30.6	36	2.7	36	0.1	<0.0001

^1^Refrigeration treatment: UNCHILLED—Data from images collected immediately after captive bolt application; CHILLED—data from images collected 24 h after captive bolt application and refrigeration at 2 to 4°C.

Soft tissue thickness caudal to the bolt path was greater (*P* = 0.0120) in UNCHILLED heads (7.2 ± 0.2 mm) than CHILLED heads (6.4 ± 0.2 mm). Soft tissue thickness rostral to the bolt path was also greater (*P* = 0.0378) in UNCHILLED heads (6.1 ± 0.1 mm) than CHILLED heads (5.5 ± 0.2 mm). Cranial thickness caudal to the bolt path was not different (*P* = 0.8659) in UNCHILLED (18.3 ± 0.6 mm) heads than CHILLED (18.1 ± 0.6 mm) heads, nor was cranial thickness rostral to the bolt path different (*P* = 0.2593) between treatments (UNCHILLED: 15.2 ± 0.6 mm; CHILLED: 16.2 ± 0.6 mm). Total tissue thickness caudal to the bolt path did not differ (*P* = 0.2577) between UNCHILLED (24.5 ± 0.6 mm) heads and CHILLED heads (24.5 ± 0.6 mm). Total tissue thickness rostral to the bolt path was also not different (*P* = 0.6730) between UNCHILLED (21.3 ± 0.7 mm) heads and CHILLED (21.7 ± 0.7 mm) heads. There was no difference (*P* = 0.0737) in exposed cross-sectional brain area between UNCHILLED (3519.9 ± 44.1 mm^2^) heads and CHILLED (3633.4 ± 44.1 mm^2^) heads. Cranial temperature was greater (*P* ˂ 0.0001) in UNCHILLED (30.6 ± 0.1°C) heads than CHILLED (2.7 ± 0.1°C) heads.

In an assessment of physical landmarks for the PCB euthanasia of cattle, Gilliam et al. (2012) concluded that the variable freezing time and 36-h thaw time may have resulted in the failure of traumatic brain injury scores, although the method they used had been validated in heads from animals that were recently shot with a PCB or bullet and not frozen. Gilliam et al. (2016) reported that that brain tissue was softened from freezing and thawing cadaver bovine heads, which resulted in the tissue collapsing into the bolt path before CT scans of the head, which may have contributed to the observation of shallow penetration depth from computerized tomography (CT) scans of heads from mature bulls.

Kline et al. (2019) reported challenges in the assessment of damage to brain tissue following the PCB stunning of cattle, due to the gelatinous nature of the brain, and recommended a 12- to 24-h chill time prior to splitting skulls in future studies for more accurate assessment of brain damage. In a related study, cattle heads were collected from a processing plant and chilled at 0°C for 24 h prior to the assessment of damage to specific regions of the brain and challenges with the assessment of brain damage were not noted (Wagner et al., 2019).

The results in our study were used to calculate a correction factor that could be used to estimate the unchilled soft tissue thickness of market pig heads at the frontal PCB site from measurements of chilled heads for use in future investigations. The following formula was used as follows: (UNCHILLED soft tissue thickness/CHILLED soft tissue thickness). The caudal and rostral soft tissue thicknesses were averaged for each head within each treatment group prior to calculation of the correction factor.

## IMPLICATIONS

As a result of the greater soft tissue thickness in UNCHILLED heads compared to CHILLED heads, a correction factor of 1.12 was determined for the prediction of UNCHILLED soft tissue thickness from CHILLED soft tissue thickness. Our results did not identify a need for a correction factor when determining UNCHILLED cranial thickness, total tissue thickness, or cross-sectional brain area from CHILLED heads. It is important to observe differences in tissue damage in future investigations of cooling effects on cadaver swine heads as this study was solely focused on detecting differences in tissue dimensions. Ultimately, the documentation of the effect of cooling on tissue dimensions of swine heads is valuable and important for future investigations of physical stunning and euthanasia methods that use cadaver heads as a model.
